# Research of Hubs Location Method for Weighted Brain Network Based on NoS-FA

**DOI:** 10.1155/2017/6174090

**Published:** 2017-06-21

**Authors:** Zhengkui Weng, Bin Wang, Jie Xue, Baojie Yang, Hui Liu, Xin Xiong

**Affiliations:** ^1^Faculty of Information Engineering & Automation, Kunming University of Science and Technology, Kunming, China; ^2^School of Communication and Information Engineering, Shanghai University, Shanghai, China; ^3^College of Information and Network Security, Yunnan Police College, Kunming, China

## Abstract

As a complex network of many interlinked brain regions, there are some central hub regions which play key roles in the structural human brain network based on T1 and diffusion tensor imaging (DTI) technology. Since most studies about hubs location method in the whole human brain network are mainly concerned with the local properties of each single node but not the global properties of all the directly connected nodes, a novel hubs location method based on global importance contribution evaluation index is proposed in this study. The number of streamlines (NoS) is fused with normalized fractional anisotropy (FA) for more comprehensive brain bioinformation. The brain region importance contribution matrix and information transfer efficiency value are constructed, respectively, and then by combining these two factors together we can calculate the importance value of each node and locate the hubs. Profiting from both local and global features of the nodes and the multi-information fusion of human brain biosignals, the experiment results show that this method can detect the brain hubs more accurately and reasonably compared with other methods. Furthermore, the proposed location method is used in impaired brain hubs connectivity analysis of schizophrenia patients and the results are in agreement with previous studies.

## 1. Introduction

Human brain is one of the most complex systems in the world. The technology of human brain reconstruction based on nuclear magnetic resonance imaging (MRI) provides a powerful tool for the study of brain structure. The construction of human brain network may be realized in three levels: microscale (neuron), small-scale (neural cluster), and large-scale (brain region) [1]. Due to the physical particularity of the human brain and the limitation of magnetic resonance (MR) data collection technology, the large-scale brain region network is still the focus of current researches, which takes the different regions of the cerebral cortex as nodes and the specific connectivity between two brain regions as the edge of the network. Those researches based on large-scale human brain network can help people to study the overall structure and operation mechanism of human brain systematically because it can take advantage of graph theory and complex network theory [2]. The brain hubs refer to the phenomenon that there exist some central nodes in the structural human brain network, which have a large number of connections with other regions and play key roles in the topology of network [1, 3]. Recent studies have indicated the importance of these hubs in brain network. A small amount of hubs plays an important role in human brain's information transmission [4], and the damage of this kind of hubs will also cause a devastating impact on the whole human brain network [5]. Researches on hubs are helpful for diagnosis and treatment of common brain diseases such as Alzheimer's disease and schizophrenia [6]; furthermore, locating hub nodes in brain network and then mapping them onto the brain corresponding anatomical regions have an important clinical value in neurosurgical operation navigation for avoiding important brain functional region impairment [7].

In the studies for location method of brain hubs, a method using rich-club connectivity coefficient has been proposed to define the hub nodes [8] and it was verified in the comparison experiment between schizophrenic patients and healthy people [9]. The hubs definition methods based on degree centrality, betweenness centrality, and closeness centrality of nodes have been used to identify the hub nodes in the human brain network and the results were analyzed to compare the effects of these three different centrality indexes [10]. Another hub detecting method has been presented from the perspective of functional regions, which regarded the nodes involved in great number of subnetworks as potential hubs and then identified the real hubs from these potential hub nodes with spatial location information [11].

Currently in the researches of cognitive science and brain diseases, the most widely used brain hubs location methods are almost based on the betweenness centrality and degree centrality. On the basis of hubs identification method with betweenness centrality, the support vector machine algorithm was used to classify the patients with schizophrenia from normal people [12]. A rich-club connection coefficient with degree centrality was defined and the connections between core brains regions of the schizophrenic patients were found to be more sparse than the normal ones because the value is obviously decreased [13]. By comparing the hubs located with betweenness centrality value, those major depression disorder patients have shown abnormal changes in structural brain network compared with healthy people [14].

The aim of this study is to develop a framework for assessing the importance of regions in structural human brain network based on T1 and DTI data. Confined to the nodes local property, most hubs location methods only use one single index such as node degree centrality or betweenness centrality to define the importance of a region in the brain network, but as a part of complex system, the global performance of a node in the whole brain network is more important than its local performance. In this work, the number of white matter streamlines (NoS) between brain regions is taken as the weight to get a weighted adjacency matrix as the original human brain network. For more precise description of brain essential biological property, anisotropic fraction (FA) value is fused to correct the weight value deviation; then a new weighted brain adjacency matrix is built which is called NoS-FA matrix in this paper. Taking account of both local and global properties of a node, the brain region importance contribution matrix and information transmission efficiency value are constructed, respectively, based on NoS-FA matrix. Finally these two factors are used together to get an importance indicator for each node and then the hubs can be located according to the value.

Three experiments have been designed and finished to verify our proposed method. The results of hubs evaluation performance contrast experiment show that this method has better distinguishability and rationality. The results of vulnerability analysis experiment exhibit that hubs obtained with this method have more distinct influence on the overall density and efficiency of the human brain network when they are impaired. When applying this method for hubs locating in schizophrenia patients, siblings, and healthy people, the experiment results indicate that reasonable differences exist in these three groups which is in accordance with previous researches. The method proposed in this work could provide a new insight into systematic analysis of brain region and it is generalizable to the researches of how to find hub nodes in other similar networks.

## 2. Reconstruction of NoS-FA Weighted Brain Structural Network

### 2.1. Workflow for Reconstruction of Human Brain Structural Network

MR data was acquired on a 1.5 tesla GE scanner using the quadrature head coil and data acquisition included anatomical DTI and T1 weighted image. Acquisition parameters for the DTI-MR are as follows: high angular gradient set of 15 different weighted directions and 1 unweighted b0 scan; TR = 11000 ms, TE = 74.7 ms; b weighting of 1000 s/mm^2^; matrix size = 128 × 128; field of view = 240 mm × 240 mm; slice thickness = 4 mm; slice gap = 0 mm; 35 slices covering the whole brain for each individual subject.

**Figure 1:**
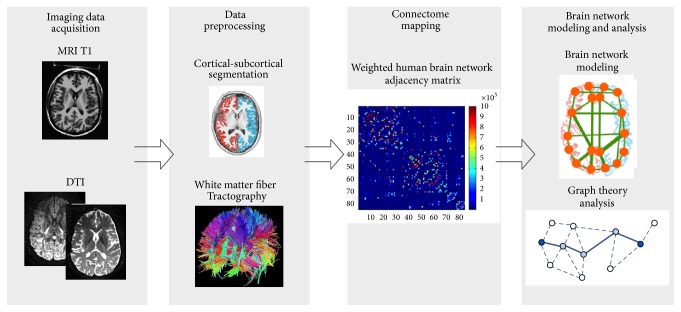
The workflow to create weighted human brain network.

Several steps are necessary to construct the human brain structural network from T1 and diffusion MRI data as illustrated in Figure 1. Firstly T1 and DTI image data need to be acquired. Then the data need to be preprocessed, including format conversion of raw data, head realignment, eddy current distortions, and other necessary processing. Segmentation of the brain in white matter, grey matter, and Cerebrospinal Fluid (CSF) with T1 weighted image needs to be performed, and on this basis the brain cortical is divided into 83 brain regions by using Automated Anatomical Labeling (AAL) template in Cortical Parcellation with FreeSurfer [15], which will serve later on as 83 nodes of the brain structural network. With DTI image data the intravoxel reconstruction of diffusion information needs to be done to get the FA quantity and the fiber tracking needs to be performed with Tractography technique to get the number of streamlines between brain regions. These can be defined as the weight coefficients of the edges in the brain structural network [16]. The connectivity matrix is obtained by registering the two image spaces (morphological and diffusion). We can use the weighted human brain network adjacency matrix to represent the weighted human brain structural network. The color of each brain region changes from blue to red in the matrix, which represents the connectivity strengths that vary from the lowest to the highest. All the steps are processed with the Connectome Mapping Toolbox [17].

### 2.2. Fusing Method of NoS-FA Weighted Matrix

When using graph theory for the research, the weighted human brain network can be expressed with an undirected graph *G* = {*V*, *E*}, which consists of *n* nodes and *m* edges. Here *V* = {*v*_1_, *v*_2_, *v*_3_,…, *v*_*n*_} represents the collection of nodes and *E* = {*e*_1_, *e*_2_, *e*_3_,…, *e*_*n*_} represents the set of edges in the human brain network.

*W* is the weighted connective matrix of the network *G* and *w*_*ij*_ is used to represent the weight value between node *i* and node *j*. Since diffusion is a symmetric process and the connection between two brain regions is regarded as undirected, *W* is a symmetric matrix; that is to say, *w*_*ij*_ = *w*_*ji*_.

(1) $$\begin{array}{c} W=\left(\begin{array}{ccc} w_{11} & \ldots & w_{1j}\\ \vdots & \ddots & \vdots \\ w_{i1} & \cdots & w_{ij} \end{array}\right). \end{array}$$

Taking the number of streamlines between brain regions *M*_*ij*_^NoS^ as the strength of connection between adjacent nodes *i* and *j*, which is always a positive value [18], the edge weight value *w*_*ij*_ of the adjacency matrix should be

(2) wij=Inf,i=j,Mij,i≠j,    i  and  j  are  connected  directly,0,i≠j,    i  and  j  are  not  connected  directly.

The number of streamlines connecting two regions is a simplistic and direct measure of connectivity, but in the process of Tractography, there exist a large number of white matter fibers crossing, convergence, and branching in a single voxel [19]. Because the size of each brain region is different, the region with larger area will access more fiber connections than the smaller ones. As a result, the numbers of streamlines between brain regions obtained by Tractography do have some deviations.

In order to reduce the influence of the deviations mentioned above, we propose a weighted adjacent matrix construction technique by fusing the fractional anisotropy index together with the number of streamlines. The fractional anisotropy value is based on the normalized variance of the eigenvalues and its range is between 0 and 1 (0 = isotropic diffusion, 1 = highly directional). As a physical characteristic of different tissues in the brain, the FA value of the same object is comparability in different time, different objects, and different imaging equipment [20, 21]. FA can give information about the shape of the diffusion tensor at each voxel and it is a kind of diffusion properties of water molecules in the brain, so it can be used to characterize the connectivity strength among each pair of brain region.

Firstly, in order to eliminate the influence of different physical variables, the FA weighted adjacent matrix *M*^FA^ is normalized as follows:

(3) $$\begin{array}{c} M_{ij}^{FA}=\frac{M_{ij}^{FA}-\min M_{ij}^{FA}}{\max M_{ij}^{FA}-\min M_{ij}^{FA}}. \end{array}$$

Then taking *M*_*ij*_^FA^ as the correction parameters, each element in *M*_*ij*_^NoS^ adjacency matrix is combined with this value so as to get a fused NoS-FA weighted adjacency matrix *M*_*ij*_^NoS-FA^ which is defined as

(4) $$\begin{array}{c} M_{ij}^{NoS\text{-}FA}=M_{ij}^{NoS}\times M_{ij}^{FA}. \end{array}$$

This network has included not only the connection strength of fibers between two connected brain regions but also the inherent physical property of each region, so it can show more comprehensive bioinformation of the brain and we will take it as the foundation of our study in this work.

## 3. Human Brain Hubs Location Method Based on NoS-FA Matrix

### 3.1. Construction of Brain Region Importance Evaluation Matrix

Just like other complex networks, the human brain network is an integration of nodes and edges, and the importance of each node will be affected by all those connections which it has. That means when change happened with even one node, it will lead to the disorder or collapse of the entire network [22]. The relationships with other brain regions have very important influence on the performance of a node, and it is not enough to describe the complexity of topological relation only by the local characteristic of the brain region. On the basis of reference [23], in which a contribution matrix of the node importance degree in undirected and unweighted networks was presented, a hub evaluation method with weighted importance contribution matrix is proposed in our work. In this method, both the contribution of a single brain region for the other connected brain regions in the whole brain network and the information transfer ability of this brain region are considered together to find the hubs in the brain network effectively.

In a brain structural network with *n* brain regions, if the average connection degree of all brain regions is $\bar{k}$, which indicates the average number of all connections in the human brain, and the average brain connection strength is $\bar{S}$, which represents the average number of white matter fibers in all brain regions, then a single brain region *v*_*i*_ will have a contribution $D_{i}/\bar{S}{\bar{k}}^{2}$ to its connected brain regions. Because the NoS-FA adjacency matrix is weighted, the contribution of each brain region *v*_*i*_ to other connected brain regions should also consider the weight value *w*_*ij*_, so the importance contribution matrix of brain regions in the human brain network *H*_BRIM_ is defined as

(5) $$\begin{array}{c} H_{BRIM}=\left[\begin{array}{cccc} 1 & \frac{D_{2}w_{21}}{\bar{S}{\bar{k}}^{2}} & \cdots & \frac{D_{n}w_{n1}}{\bar{S}{\bar{k}}^{2}}\\ \frac{D_{1}w_{12}}{\bar{S}{\bar{k}}^{2}} & 1 & \cdots & \frac{D_{n}w_{n2}}{\bar{S}{\bar{k}}^{2}}\\ \vdots & \vdots & \cdots & \vdots \\ \frac{D_{1}w_{1n}}{\bar{S}{\bar{k}}^{2}} & \frac{D_{2}w_{2n}}{\bar{S}{\bar{k}}^{2}} & \cdots & 1 \end{array}\right]. \end{array}$$

Here the diagonal elements have a contribution value of 1.

On the other hand, in order to reflect the ability of a single brain region *v*_*i*_ in the information process, the information transmission efficiency *E*_*i*_^*w*^ is defined as

(6) $$\begin{array}{c} E_{i}^{w}=\frac{1}{n}\overset{}{\underset{i\in n}{\sum }}\frac{\sum_{j,h\in n,j\neq i}^{}a_{ij}a_{jh}{\left(d_{ij}^{w}\right)}^{-1}}{D_{i}\left(D_{i}-1\right)}. \end{array}$$

Here *a*_*ij*_ is used to indicate whether there is a direct link between nodes *i* and *j*, if the connection exists, *a*_*ij*_ = 1; otherwise, *a*_*ij*_ = 0. *d*_*ij*_^*w*^ represents the shortest weighted distance between two different brain regions, which is the harmonic mean weight of each brain region.

(7) dijw=min11/wik+⋯+1/wnj,11/wiv+⋯+1/wwj,…,11/wil+⋯+1/wkj.

It can be seen from the definition of *E*_*i*_^*w*^ that the transmission efficiency can reflect how important a brain region is in the information transfer process in human brain. If *E*_*i*_^*w*^ value of a brain region is very big that means it plays a more important role in information transmission; therefore, when this brain region is injured, the information transmission ability of the whole brain network will suffer a greater loss.

By now for each brain region we have a local contribution index $D_{i}\omega_{ij}/\bar{S}{\bar{k}}^{2}$ and a global importance property index *E*_*i*_^*w*^; then the values of these two index are integrated into an evaluation matrix *H*_RC_ as follows:

(8) $$\begin{array}{c} H_{RC}=\left[\begin{array}{cccc} E_{1}^{w} & \frac{D_{2}w_{21}}{\bar{S}{\bar{k}}^{2}}E_{2}^{w} & \cdots & \frac{D_{n}w_{n1}}{\bar{S}{\bar{k}}^{2}}E_{n}^{w}\\ \frac{D_{1}w_{12}}{\bar{S}{\bar{k}}^{2}}E_{1}^{w} & E_{2}^{w} & \cdots & \frac{D_{n}w_{n2}}{\bar{S}{\bar{k}}^{2}}E_{n}^{w}\\ \vdots & \vdots & \cdots & \vdots \\ \frac{D_{1}w_{1n}}{\bar{S}{\bar{k}}^{2}}E_{1}^{w} & \frac{D_{2}w_{2n}}{\bar{S}{\bar{k}}^{2}}E_{2}^{w} & \cdots & E_{n}^{w} \end{array}\right]. \end{array}$$

Here *H*_RC_(*i*, *j*) indicates the important influence of brain region *i* to brain region *j*, which depends not only on the quantity of white matter fibers between *i* and *j* but also on the important level of brain region *i* in the information transmission. Through the application of the hubs evaluation matrix *H*_RC_, the important index of brain regions is expressed as follows:

(9) $$\begin{array}{c} {RC}_{i}=E_{i}^{w}\times \frac{\sum_{j=1,j\neq i}^{n}D_{j}w_{ji}E_{j}^{w}}{\bar{S}{\bar{k}}^{2}}. \end{array}$$

We can calculate all the RC_*i*_ values in the brain network and choose the 15 highest values of the brain regions as hubs in a human brain network [24].

### 3.2. Workflow of Hubs Location Based on Important Index *RC*_*i*_

**Figure 2:**
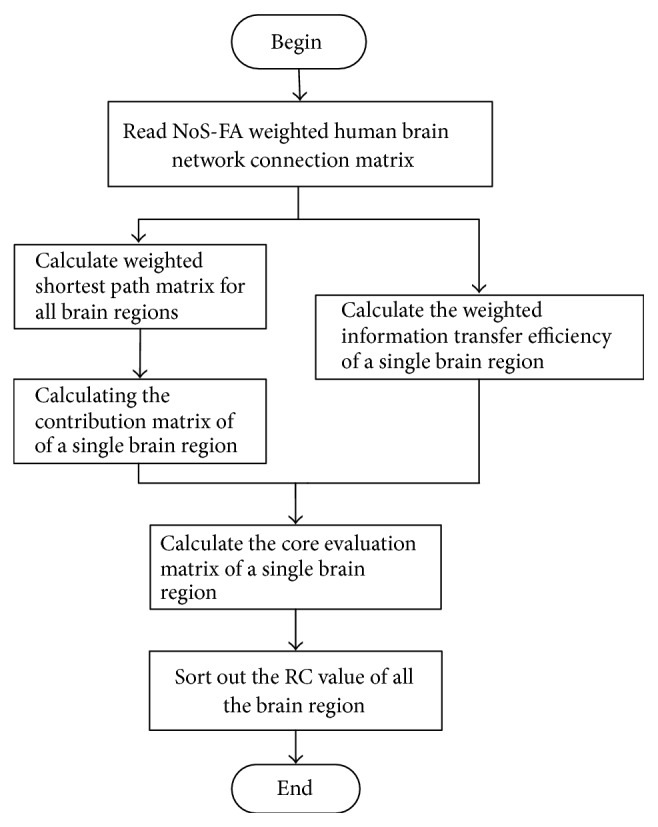
The workflow of weighted human brain network hubs location process.

The brain hubs location algorithm based on important index considers both the global property of a brain region and the relationships with other connected regions in the brain; the overall workflow is as follows:

1. The quantity of white matter fiber between regions is fused with anisotropic fraction value of this region to get *M*^NoS-FA^ according to (4), which is used as the input data of the algorithm.
2. The importance matrix of all brain regions to the other connected brain regions is computed, respectively, according to (5).
3. The information transfer efficiency values of each region are computed with (6) and integrated into the relative importance matrix.
4. RC_*i*_ value is calculated according to (8) and (9), which represents the importance of each brain region.
5. Ranking the RC_*i*_ values in descending order, the nodes with the first 15 highest values are considered as hubs in brain structural network.
6. The algorithm flow diagram is shown as Figure 2.

## 4. Effect Analysis of Hubs Location Algorithm Based on NoS-FA Matrix

### 4.1. Effectiveness Analysis of the Algorithm

**Figure 3:**
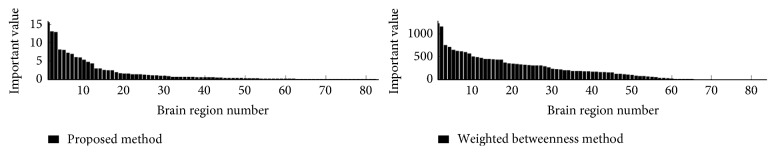
Comparison of the region evaluation results with different methods.

We use both the weighted betweenness hubs location method which is the most popular method in evaluating brain hubs and proposed hubs location method to calculate the importance value of each brain region for the same healthy people's brain network, and the results were shown in Figure 3.

There are three improvements when using the proposed method. Firstly, from the results, we can see when using the region evaluation method with weighted betweenness that there exist some nodes that have the same importance value. While in the results based on the proposed method every region has different value of importance, so our method is more accurate for evaluating the importance of brain regions. In addition, it can be seen clearly in the region evaluation results with weighted betweenness method that some brain regions have the same importance value of zero, but it is impossible for a region to have no importance in the network. While in the results of proposed method even the last one also has a no-zero importance value, so our method is more reasonable for evaluating the importance of brain regions. Finally in the region evaluation results with weighted betweenness method, the distribution of importance value is more even and the values in different regions are closed to each other. While in the results with proposed method the distribution looks sharper, the difference between the hubs and other noncore nodes in the brain network is more apparent. For further analysis, the most important 15 brain regions located with two methods and some corresponding properties are listed in sort order in Table 1.

**Figure 4:**
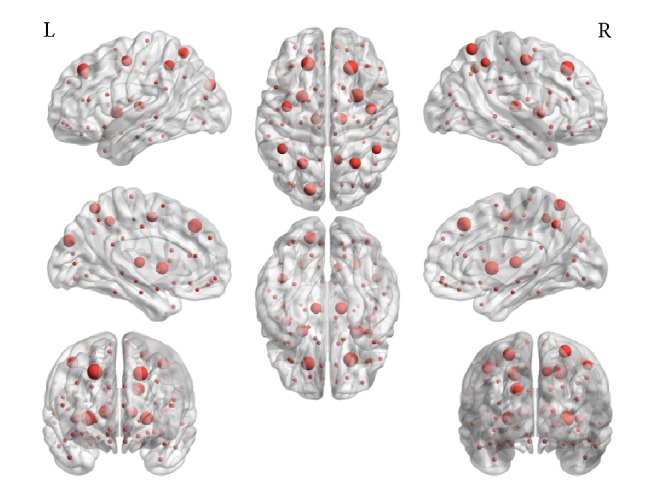
Location map of hubs in weighted human brain network based on NoS-FA.

The location of hubs with the proposed method in brain space is shown in Figure 4; it can be seen that the human brain hubs are mainly located on the frontal lobe, parietal lobe, and the flat layer part of the organization, including the superior parietal gyrus, parietal gyrus, superior frontal gyrus, precentral gyrus, paracentral gyrus, thalamus, putamen, and brain stem. Benefitting from fusing two kinds of bioinformation, the NoS-FA weighted network involves more comprehensive information of brain, so it can distinguish the hub nodes from those noncore nodes more accurately; at the same time, the ranking of hubs importance value is more reasonable and highly recognizable.

### 4.2. Algorithm Vulnerability Analysis and Comparison

In order to verify the actual importance of the hubs, which are located by the proposed algorithm, the vulnerability analysis experiment is presented in our work. When a node in the network is removed, the global property of the network will be changed. Usually the ratio of the change of network property to the network property before the removal is defined as the vulnerability [25]. The greater vulnerability a node has, the higher damage will be put on the entire network, and the role of this node is more important. It needs to be emphasized that, in the experiment, when one node is removed, all the white matter fibers, which are connected with this brain regions, are invalid and the NoS weight of related edges in the network will be zero. Therefore, the vulnerability of brain region *i* is defined as

(10) $$\begin{array}{c} V_{i}=\frac{PropValue-{PropValue}^{\prime }}{PropValue}. \end{array}$$

Here PropValue represents one kind of network properties value before a node is removed, and PropValue′ represents this property value after the removal. In this research, the network properties of global efficiency and network density are taken as the vulnerability analysis parameters, respectively, which are shown as

(11) DensityValue=∑i=1,j=1nMijwij≠0nn−1,EfficiencyRating=1n∑i∈n∑j∈n,j≠idijw−1n−1.

The network density reflects the ratio of the actual number of edges and the maximum number of edges the network may have, and it is an important attribute to test the network size. The global efficiency reflects the information transmission capability of a network.

**Figure 5:**
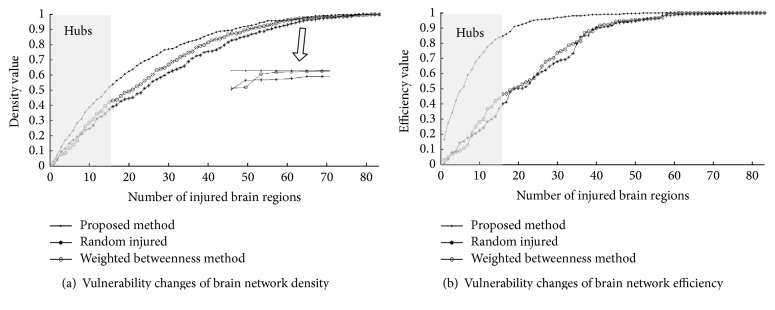
Vulnerability change comparison of brain network vulnerability.

To compare the influence that a hub may have on the entire brain network, three cases are considered in this experiment: brain hubs are computed and chosen with our proposed method, with weighted betweenness method or at random. The vulnerability change curves of three methods are given and compared as shown in Figure 5. It can be seen that when one brain region is damaged, the properties of the whole brain network have also changed and the hubs obtained by proposed method have the greatest impact on the overall properties of the human brain network in both density and efficiency. Because both the local characteristic of a single brain region and the global contribution that a brain region has to its connected regions are considered together, the evaluation process is based on more comprehensive bioinformation and so the hubs located with this method will have more important influence on the human brain network.

### 4.3. Hubs Property Analysis of Schizophrenia

The proposed method was applied to the MRI data of schizophrenia for the analysis of human brain structural network changing. Total of 205 people were divided into three groups: schizophrenia patients group (Patients) with 62 people, siblings of patients (Siblings) with 83 people, and healthy people group (healthy people) with 60 people. All the MRI data were processed according to the workflow in Figure 1.

Firstly three types of global brain network properties were calculated, respectively, to make the comparison. (1) Brain region connection strength *S* = (1/*N*)∑_*j*∈*N*_*a*_*ij*_, which is the mean value of the weight values of all connections in the weighted brain network. The connection strength is the direct reflection of the numbers of white matter fibers in the human brain network. (2) The global efficiency *E* = (1/*N*)∑_*i*=1,*i*≠*k*_^*n*^(1/*d*_*ki*_) of the brain network, which is the mean of all the reciprocals of the shortest path. Efficiency value reflects the transmission speed of information in the human brain network. (3) Clustering coefficient *C* = (1/*N*)∑_*i*∈*v*_*C*_*i*_ of the brain network, which is the mean value of all clustering coefficients of all regions. Clustering coefficient is a measure of the degree of brain network group indicating the extent of the network clustering.

**Figure 6:**
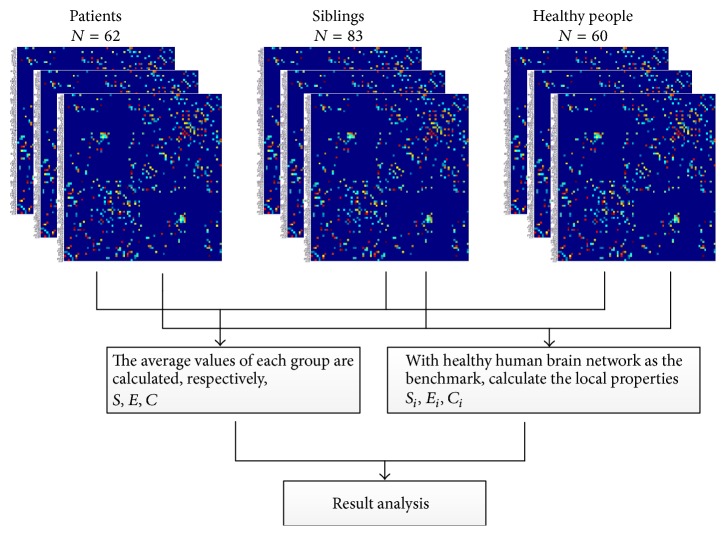
Experiment process diagram.

Taking the healthy human brain network as the benchmark, three local property values of each hub were calculated for each subject: connection strength *S*_*i*_ = ∑_*j*∈*N*_*a*_*ij*_, local efficiency *E*_*i*_ = ∑_*i*=1,*i*≠*k*_^*n*^(1/*d*_*ki*_), and clustering coefficient *C*_*i*_ = 2*E*_*i*_/*k*_*i*_(*k*_*i*_ − 1); then the average values in each groups were calculated, respectively. The experiment is designed as the process shown in Figure 6 and all the computations are executed by the MATLAB brain connectivity toolbox [26].

The experiment results are shown in Table 2. The average global properties values of the brain network are presented in Table 2(a); the average local properties values with weighted betweenness method are presented in Table 2(b) and the average local properties values with proposed method are presented in Table 2(c). It can be seen from the results in Table 2 that both global and local properties in patients, siblings, and healthy people group have shown some interesting differences.

The global properties values are shown in Table 2(a), and compared with healthy group, the average values of connection strength, global efficiency, and clustering coefficient in patients group were decreased by 3.95%, 2.69%, and 3.55%, respectively. The significant ordered differences, such that healthy people > siblings > patients, were found in both connection strength and clustering coefficient, while for global efficiency patient group has a weak higher value than sibling group but still lower than healthy group.

The local properties values based on weighted betweenness method are shown in Table 2(b). The sequence that healthy people > siblings > patients still can be found but not so obvious in connection strength and clustering coefficient between three groups. The local efficiency of patient group is very close to that of sibling group, but the former was slightly higher than the latter.

For the local properties values based on the proposed method in Table 2(c), the significant ordered difference is that healthy people > siblings > patients were found clearly in connection strength, clustering coefficient, and local efficiency. The average values of hubs' local efficiency were the highest in healthy people, intermediate in siblings (4.36% reduced relative to healthy people), and lowest in patients (8.44% reduced compared with healthy people). Connection strength in patients hubs was decreased by 9.72% compared with healthy people and 6.55% compared with siblings. Clustering coefficients in patients hubs were decreased by 8.47% compared with healthy people and 4.53% compared with siblings.

These results are consistent with the conclusion in [13]. More importantly, from the results of the above analysis, we can see that the difference between the hubs of healthy people, siblings, and patients in the proposed method is more apparent than in weighted betweenness method. These results indicate that hubs location with method proposed in this paper is more reasonable and accurate than the weighted betweenness method, which is most popular in finding hubs at present researches.

At the meantime, analysis of variance (ANOVA) was finished in our work to test the data difference and the results are shown in Table 3. The *F* value of connection strength *S*_*i*_, clustering coefficient *C*_*i*_, and local efficiency *E*_*i*_ was 8.496, 5.325, and 5.864, and the *P* value of each group was less than 0.05.

## 5. Conclusions

In this work, we presented a novel hub location method for the human brain structural network, which is based on MRI image reconstruction technique. One meaningful work is that the NoS weights matrix was fused with FA values to get more comprehensive bioinformation for brain region connections. The other valuable work is the construction of contribution matrix of the region's importance, which is an index including local contribution of a region to other correlative brain regions and the global transmission efficiency of this region in NoS-FA weighted human brain network. The experiment results testify that the proposed method can provide more precise and reasonable hubs location method compared with the most frequently used weighted betweenness evaluation index. The experiment results also emphasize the findings discovered by other researches; the hubs of human brain network in schizophrenia patients are impaired compared with healthy people.

## Figures and Tables

**Table 1:** Comparison of the evaluation results under different methods.

Hub order number	Weighted betweenness method			Proposed method		
	Importance value	Region number	region name	Importance value	Region number	Region name
1	1234	12	R-RAC	15.515	8	R-SF
2	1162	56	L-ISTC	13.099	49	L-SF
3	748	32	R-ST	12.868	37	R-PUT
4	714	63	L-PCAL	8.070	78	L-PUT
5	644	62	L-CUN	7.987	83	BS
6	622	42	L-LOF	7.204	35	R-THA
7	616	15	R-ISTC	6.942	18	R-SP
8	598	37	R-PUT	5.972	10	R-PREC
9	568	59	L-SMAR	5.892	59	L-SP
10	498	36	R-CAU	5.315	76	L-THA
11	482	55	L-PC	4.693	51	L-PREC
12	472	35	R-THA	4.346	60	L-IP
13	448	25	R-FUS	2.961	19	R-IP
14	448	34	R-INS	2.942	20	R-PCUN
15	436	71	L-MT	2.509	34	R-INS

**Table:** (a) Global properties values of the human brain network

Experimental group	Connection strength	Global efficiency	Clustering coefficient
Healthy people	9261.2	224.27	134.74
Siblings	9030.7	214.34	127.25
Patients	8895.3	218.24	122.49

**Table:** (b) Local properties values of hubs based on weighted betweenness method

Experimental group	Connection strength	Local efficiency	Clustering coefficient
Healthy people	14491	231.57	102.92
Siblings	14188	221.82	99.95
Patients	14170	222.58	98.82

**Table:** (c) Local properties values of hubs based on the proposed method

Experimental group	Connection strength	Local efficiency	Clustering coefficient
Healthy people	28097	410.74	189.37
Siblings	27142	392.82	181.56
Patients	25365	376.04	173.33

**Table:** (a) Variance analysis result of connection strength *S*_*i*_

	Sum of square	Degree of freedom	Mean of square	*F*	Sig.
Between-group	2.369*E*8	2	1.185*E*8	8.496	.000
Intragroup	2.817*E*9	202	13943176.62		
Total	3.053*E*9	204			

**Table:** (b) Variance analysis result of clustering coefficient *C*_*i*_

	Sum of square	Degree of freedom	Mean of square	*F*	Sig.
Between-group	7851.000	2	3925.500	5.325	.006
Intragroup	148924.522	202	737.250		
Total	156775.522	204			

**Table:** (c) Variance analysis result of local efficiency *E*_*i*_

	Sum of square	Degree of freedom	Mean of square	*F*	Sig.
Between-group	36705.578	2	18352.789	5.864	.003
Intragroup	632256.889	202	3129.985		
Total	668962.467	204			

## References

[1] Rubinov M., Sporns O. (2010). Complex network measures of brain connectivity: Uses and interpretations. *NeuroImage*.

[2] Jinqing F. (2012). Exploring progress on brain networks (I): research characteristics, methods and three major types. *Chinese Journal of Nature*.

[3] Zhou S., Mondragón R. J. (2004). The rich-club phenomenon in the internet topology. *IEEE Communications Letters*.

[4] Daianu M., Jahanshad N., Nir T. M. (2015). Rich club analysis in the Alzheimer's disease connectome reveals a relatively undisturbed structural core network. *Human Brain Mapping*.

[5] Colombo M., Sporns O. (2014). Discovering the human connectome. *Minds and Machines*.

[6] Colombo M., Sporns O. (2013). Networks of the brain. *Minds and Machines*.

[7] Harriger L., van den Heuvel M. P., Sporns O. (2012). Rich Club Organization of Macaque Cerebral Cortex and Its Role in Network Communication. *PLoS ONE*.

[8] Van den Heuvel M. P., Sporns O. (2011). Rich-club organization of the human connectome. *The Journal of Neuroscience*.

[9] Sporns O., Honey C. J., Kötter R. (2007). Identification and classification of hubs in brain networks. *PLoS ONE*.

[10] GeethaRamani R., Sivaselvi K. Human brain hubs (provincial and connector) identification using centrality measures.

[11] Power J., Schlaggar B., Lessov-Schlaggar C., Petersen S. (2013). Evidence for hubs in human functional brain networks. *Neuron*.

[12] Cheng H., Newman S., Goñi J. (2015). Nodal centrality of functional network in the differentiation of schizophrenia. *Schizophrenia Research*.

[13] Collin G., Kahn R. S., De Reus M. A., Cahn W., Van Den Heuvel M. P. (2014). Impaired rich club connectivity in unaffected siblings of schizophrenia patients. *Schizophrenia Bulletin*.

[14] Korgaonkar M. S., Fornito A., Williams L. M., Grieve S. M. (2014). Abnormal structural networks characterize major depressive disorder: a connectome analysis. *Biological Psychiatry*.

[15] Tzourio-Mazoyer N., Landeau B., Papathanassiou D. (2002). Automated anatomical labeling of activations in SPM using a macroscopic anatomical parcellation of the MNI MRI single-subject brain. *NeuroImage*.

[16] da Silva A. R. F. (2014). Generalized diffusion tractography based on directional data clustering. *Studies in Computational Intelligence*.

[17] Daducci A., Gerhard S., Griffa A. (2012). The connectome mapper: an open-source processing pipeline to map connectomes with MRI. *PLoS ONE*.

[18] Liu T., Zeng-Ru D., Hong D. (2011). Effect of distribution of weight on the efficiency of weighted networks. *Acta Physica Sinica*.

[19] Zhang Y., Song Z.-J. (2014). Advance in the study of white matter tractography. *Fudan University Journal of Medical Sciences*.

[20] Feng W. S. (2013). Clinical outcome prediction of the DTI quantification of spinal cord with high intensity signal on 3.0T MRI in Cervical Spondylotic Myelopathy and corresponding pathological mechanism.

[21] Guang-wu H., Yuan-xiang L., Tian-zhen S. (2006). Study development of MR diffusion tensor imaging on brain. *Chinese imaging journal of integrated traditional and western medicine*.

[22] Zhang K., Li P., Zhu B., Hu M. (2013). Evaluation method for node importance in directed-weighted complex networks based on PageRank. *Journal of Nanjing University of Aeronautics and Astronautics*.

[23] Xuan Z., Feng-ming Z., Ke-wu L. (2012). Finding vital node by node importance evaluation matrix in complex networks. *Acta Physica Sinica*.

[24] Ball G., Aljabar P., Zebari S. (2014). Rich-club organization of the newborn human brain. *Proceedings of the National Academy of Sciences of the United States of America*.

[25] Pan C., Xiao-feng W., Yi L. (2010). Attack strategy for uncertain topology of complex networks. *Application Research of Computers*.

[26] Rubinov M., Kötter R., Hagmann P., Sporns O. (2009). Brain connectivity toolbox: a collection of complex network measurements and brain connectivity datasets. *NeuroImage*.

